# Longitudinal dietary trajectories from preconception to mid-childhood in women and children in the Southampton Women’s Survey and their relation to offspring adiposity: a group-based trajectory modelling approach

**DOI:** 10.1038/s41366-021-01047-2

**Published:** 2021-12-16

**Authors:** Kathryn V. Dalrymple, Christina Vogel, Keith M. Godfrey, Janis Baird, Nicholas C. Harvey, Mark A. Hanson, Cyrus Cooper, Hazel M. Inskip, Sarah R. Crozier

**Affiliations:** 1 MRC Lifecourse Epidemiology Centre, University of Southampton, Southampton General Hospital, Southampton, UK; 2 Department of Women and Children’s Health, School of Life Course Sciences, King’s College London, London, UK; 3 NIHR Southampton Biomedical Research Centre, University of Southampton and University Hospital Southampton NHS Foundation Trust, Southampton, UK; 4 NIHR Applied Research Collaboration Wessex, Southampton Science Park, Innovation Centre, 2 Venture Road, Chilworth, Southampton, SO16 7NP UK; 5 Institute of Developmental Sciences, Faculty of Medicine, University of Southampton, Southampton, UK; 6 NIHR Oxford Biomedical Research Centre, University of Oxford, Oxford, UK

**Keywords:** Risk factors, Obesity

## Abstract

**Background:**

Rates of childhood obesity are increasing globally, with poor dietary quality an important contributory factor. Evaluation of longitudinal diet quality across early life could identify timepoints and subgroups for nutritional interventions as part of effective public health strategies.

**Objective:**

This research aimed to: (1) define latent classes of mother-offspring diet quality trajectories from pre-pregnancy to child age 8–9 years, (2) identify early life factors associated with these trajectories, and (3) describe the association between the trajectories and childhood adiposity outcomes.

**Design:**

Dietary data from 2963 UK Southampton Women’s Survey mother-offspring dyads were analysed using group-based trajectory modelling of a diet quality index (DQI). Maternal diet was assessed pre-pregnancy and at 11- and 34-weeks’ gestation, and offspring diet at ages 6 and 12 months, 3, 6-7- and 8–9-years using interviewer-administered food frequency questionnaires. At each timepoint, a standardised DQI was derived using principal component analysis. Adiposity age 8–9 years was assessed using dual-energy X-ray absorptiometry (DXA) and BMI *z*-scores.

**Results:**

A five-trajectory group model was identified as optimal. The diet quality trajectories were characterised as stable, horizontal lines and were categorised as poor (*n* = 142), poor-medium (*n* = 667), medium (*n* = 1146), medium-better (*n* = 818) and best (*n* = 163). A poorer dietary trajectory was associated with higher maternal pre-pregnancy BMI, smoking, multiparity, lower maternal age and lower educational attainment. Using linear regression adjusted for confounders, a 1-category decrease in the dietary trajectory was associated with higher DXA percentage body fat (0.08 SD (95% confidence interval 0.01, 0.15) and BMI *z*-score (0.08 SD (0.00, 0.16) in the 1216 children followed up at age 8–9 years.

**Conclusion:**

Mother-offspring dietary trajectories are stable across early life, with poorer diet quality associated with maternal socio-demographic and other factors and childhood adiposity. The preconception period may be an important window to promote positive maternal dietary changes in order to improve childhood outcomes.

## Introduction

Rates of childhood obesity are increasing worldwide, with significant short- and long-term adverse health consequences [1]. Globally, 124 million school-age children and adolescents are estimated to have obesity, a substantial rise from 11 million in the 1970s [2]. The UK is no exception, with high rates of childhood obesity. Recent figures from the National Child Measurement Programme show that nearly a quarter of children under the age of five are overweight or obese. This statistic increases to a third by the time children enter secondary school [3]. Furthermore, longitudinal modelling suggests that once obesity is established in early life it tracks through adolescence to adulthood [4], thus creating a life-long condition [5]. Since the determinants of obesity are critically important for population health, there has been a major research effort in recent years to identify the responsible contributing factors in order to reduce rates of childhood obesity [6]. The strategy published by the World Health Organization (WHO) Commission on Ending Childhood Obesity (ECHO) Report concludes that reducing rates of childhood obesity requires identifying critical lifecourse periods when intervention may be most effective, including in the mother before conception and in pregnancy before conception [7].

Poor diet quality is recognised as an important contributing factor in the development of childhood obesity [8] and this association is evident even before conception [9]. Several studies have independently reported relationships between childhood obesity and maternal antenatal diet [10], breastfeeding duration [11], the development of child eating behaviours [12] and a high intake of processed and sweet foods [13]. Additionally, diet quality is known to vary during early life as it is influenced by family and environmental exposures [14] so single assessment timepoints may not reflect changes in diet intake over time. In addition, while several methods are available for modelling longitudinal data [15] these techniques have not as yet been applied to diet quality data in early life. Modelling dietary trajectories and classifying individuals into subgroups (latent classes) could identify timepoints and subgroups at risk of poor dietary intake, therefore providing important information about when and in whom to intervene with nutritional interventions. Such modelling of dietary data could provide valuable insight into the influence of poor diet quality on health outcomes and inform future public health strategies focused on prevention of childhood obesity.

In accordance with the recommendation in the WHO ECHO Report we have modelled latent class trajectories of longitudinal diet quality index (DQI) data across early life and examined the association of these trajectories with childhood obesity outcomes using data from the UK Southampton Women’s Survey (SWS), the only population-based longitudinal study in Europe of women and their children for which information was obtained from the mothers before the conception of the child [16]. The aim of this present analysis was to: (1) define latent classes of diet quality trajectories from pre-pregnancy to 8–9 years of age using group-based trajectory modelling of a DQI, (2) identify early life sociodemographic variables associated with these trajectories, and (3) describe the association between the trajectories and adiposity outcomes in 8–9-year-old children obtained from dual-energy X-ray absorptiometry (DXA), BMI *z*-scores and mid-upper arm and waist circumferences.

## Methods

### Participants

This study is a longitudinal analysis from the SWS, a cohort of women and their children living in the city of Southampton, UK [16]. The initial wave of the study was designed to measure the pre-pregnancy characteristics (lifestyle, diet and anthropometry) of women aged 20–34 years. Of those who subsequently became pregnant, follow-ups were performed across pregnancy, infancy and childhood. Between April 1998 and December 2002, 12,583 initially non-pregnant women were recruited to the SWS. The women who subsequently became pregnant were invited to attend face to face follow-ups at 11, 19- and 34-weeks’ gestation; 3158 had liveborn singleton infants in the study. The offspring have been studied across infancy (birth, 6 and 12 months) and childhood (2, 3, 4, 6–7 and 8–9). All interviews were performed by trained research nurses. SWS was conducted according to the guidelines laid down in the Declaration of Helsinki and was approved by the Southampton and South West Hampshire Local Research Ethics Committee (08/H0502/95). Written informed consent was obtained from all participating women and by a parent or guardian with parental responsibility on behalf of their children.

### Maternal data

At the preconception interview, details of maternal ethnicity, highest educational attainment (defined in six groups), smoking status and parity were recorded. Height was measured with a portable stadiometer (Harpenden; CMS Weighing Equipment Ltd, London, UK) to the nearest 0.1 cm with the head in the Frankfort plane. Weight was measured with calibrated electronic scales (Seca, Hamburg, Germany) to the nearest 0.1 kg (after removal of shoes, heavy clothing and jewelry). The dominant household social class (by occupation) was also defined (according to the Registrar General classifications). For those women who became pregnant, any smoking in pregnancy was derived (yes vs. no) from the smoking questionnaires before, during and after pregnancy. Maternal age was calculated at birth.

### Childhood data

At birth, the baby was weighed on calibrated digital scales to the nearest 1 g (Seca) and gestational age calculated from the expected delivery date, estimated by the date of the last menstrual period or obstetric ultrasound. At the 6- and 12-month visits duration of breastfeeding was defined according to the date of the last breastfeed [17]. When the child was age 8–9 years, height was recorded using a portable stadiometer to the nearest 0.1 cm (Leicester height measurer) and weight using calibrated digital scales to the nearest 0.1 kg (Seca). BMI *z*-scores were calculated using the WHO reference data, which are age and sex standardised [18]. Anthropometry was also assessed by mid-upper arm and waist circumferences (measured in triplicate and the mean derived). The children who completed the 8–9-year visit (*n* = 1216) were also invited to undergo a DXA scan to assess body composition [Hologic Discovery A machine (Hologic Inc., Bedford, MA, USA; Software V12.7.3)]. Total and proportionate fat mass were derived from the whole-body scan through the use of pediatric software. All scan results were checked independently by two trained operators. The coefficient of variation for body composition analysis using the DXA instrument was 1.4–1.9%.

### Food frequency questionnaires (FFQ)

Age specific FFQs were used to measure diet quality among women and their offspring. These measures aimed to capture information about habitual dietary intake and covered an age-appropriate time period. Maternal dietary intake was recorded at the preconception, 11- and 34-weeks’ gestation visits [19]. In the mother, food intake over the previous 3-months was assessed using a 100-item validated FFQ [19]. Dietary intake was assessed in the offspring using age-specific FFQs when they were aged 6 and 12 months and 3, 6–7 and 8–9 years of age [20–22]. In the offspring, at ages 6 and 12-months, food intake was assessed over the previous 7 days using a 34-item FFQ [21] and over the previous 4 weeks using a 78-item FFQ [22], respectively. At ages 3, 6–7 and 8–9 years, food intake was evaluated over the preceding 3 months. At the 3 and 6–7 year visits, diet was assessed using an 80-item FFQ [20]. Due to time restrictions at the 8–9 years visit, a shortened (33 question) version of the 80-item FFQ was developed; the questions were selected based on evidence of an association between certain food groups and adiposity [23] and foods found to be discriminatory on a dietary quality score [24]. Offspring questionnaires were administered by trained research nurses to the child’s parent or guardian. At each timepoint the foods listed in each FFQ were categorised into groups based on similar nutritional composition and principal component analysis was performed on the reported weekly frequencies of consumption of the food groups. At each timepoint the first principal component was found to describe a ‘DQI’ where healthy foods recommended by government guidelines were eaten frequently and less healthy foods which contribute to diet-related disease were eaten infrequently. The DQI has also been referred to as a prudent diet score [25] and an infant guidelines score [26]; participants following this dietary pattern had higher scores while those showing the opposite pattern had low scores. At each time the scores were transformed (Fisher–Yates) to a mean of zero and a standard deviation (SD) of one [27]. Given the different ways in which the data were collected over time, standardisation was important to ensure that the indices would not be on arbitrary and inconsistent scales. Full details of these analyses, included validation of the FFQs, have been published previously [20–22, 28, 29].

### Statistical analysis

For demographic statistics, binary and categorical variables are presented using counts and percentages. The distributions of continuous variables were assessed using coefficients of skewness and then summarised by mean and SD for normally distributed variables or median and interquartile range (IQR) for non-normally distributed variables.

### Trajectory modelling

Group-based trajectory modelling (GBTM) was used to identify latent classes of the dietary trajectories (traj command in Stata version 15.0). This method is a semi-parametric mixture model in which the error variance is assumed to be the same for all classes and all time points [30]. GBTM handles missing data by fitting the model using maximum likelihood estimation, and therefore handles missing data under the missing at random assumption [31]. We used a censored normal model which is appropriate for continuous normally distributed data. For GBTM, we cannot assume that all trajectories follow the same longitudinal change in diet quality. Therefore, when fitting GBTM the shape of trajectory can be modelled as either intercept, linear, quadratic and cubic. To ensure model parsimony, non-significant cubic and quadratic terms were removed from trajectories. However, linear parameters were retained irrespective of significance as long as the BIC was lower than if an intercept parameter was used [32]. This process is then repeat until there is no improvement in the Bayesian Information Criterion which assesses model fit.

For the assessment time points, these were converted to mean age from birth (e.g. late pregnancy [34 weeks’ gestation] for a full-term birth at 40 weeks was coded as -6 weeks). This study adheres to the Guidelines for Reporting on Latent Trajectory Studies (GRoLTS) checklist [33] (Supplementary Table 1); following the guidelines in the checklist, as the number of latent classes is unknown, we used a forward modelling approach from one to six classes. After fitting the one-class model, we incrementally added extra classes and investigated the model fit criteria discussed below. To identify the appropriate number of classes, each iteration (model) was assessed using likelihood-based statistics and statistics on classifying individuals.

### Likelihood-based and classification statistics

The Bayesian Information Criteria (BIC) is a test statistic for model selection and a value closer to zero indicates a better model fit. Relative entropy is a measure of model classification uncertainty and should be close to one [15]. The Average Posterior Probability Assignment (APPA) and the ratio of the Odds of a Correct Classification (OCC) are class specific. The APPA is calculated as the average posterior probability of belonging to a class over all the individuals assigned to class. The OCC is the ratio of the odds of classifying participants into number of classes for that model and is based on the maximum probability classification rule. For each class within a model, the APPA should be >70% and the OCC should be >5 [15]. Finally, the minimum proportion of participants assigned to each class should be 5% [34]. The optimal number of classes selected was based on the lowest BIC and satisfactory values for the remaining criteria.

### Association with early life demographic variables

Once the optimal number of trajectories was identified, ordered logistic regression was used to examine the relationships between the early life demographic characteristics and the trajectories. These results are reported as odds ratios (OR) with the most common group as the reference group. The early life demographic characteristics were: maternal BMI (continuous and WHO categories), highest education attainment (defined in six groups according to highest academic qualification), ethnicity (White vs. other), dominant family social class (6 categories), ever smoked and smoked in pregnancy (any vs. none), parity (0 vs. 1 + ), age at birth, breastfeeding duration (never tried, <1 month, 1–3 months, 4–6 months, 7–11 months and 12 months or more), gestational age at delivery, birthweight and sex.

### Association with adiposity outcomes at 8-years of age

Unadjusted and adjusted regression analyses were used to assess the relationships between the adiposity outcomes at 8-years of age and the dietary trajectories. Outcomes included: WHO BMI *z*-scores, arm and waist circumferences, lean mass and fat mass obtained by DXA. To identify the appropriate confounders, direct acyclic graphs were created for the offspring outcomes. These outcomes were grouped into two categories, (1) adiposity and lean mass, and (2) height. For both models the identified minimum adjustment sets were maternal BMI, parity, highest maternal education attainment and maternal age (Supplementary Figs. 1–2). To ensure that any sex and age differences were accounted for, the circumferences and DXA outcomes were also adjusted for child sex and age at the 8–9-year visit. The outcomes were also transformed to Fisher–Yates *z*-scores to allow the effect sizes to be compared [35].

## Results

**Fig. 1:**
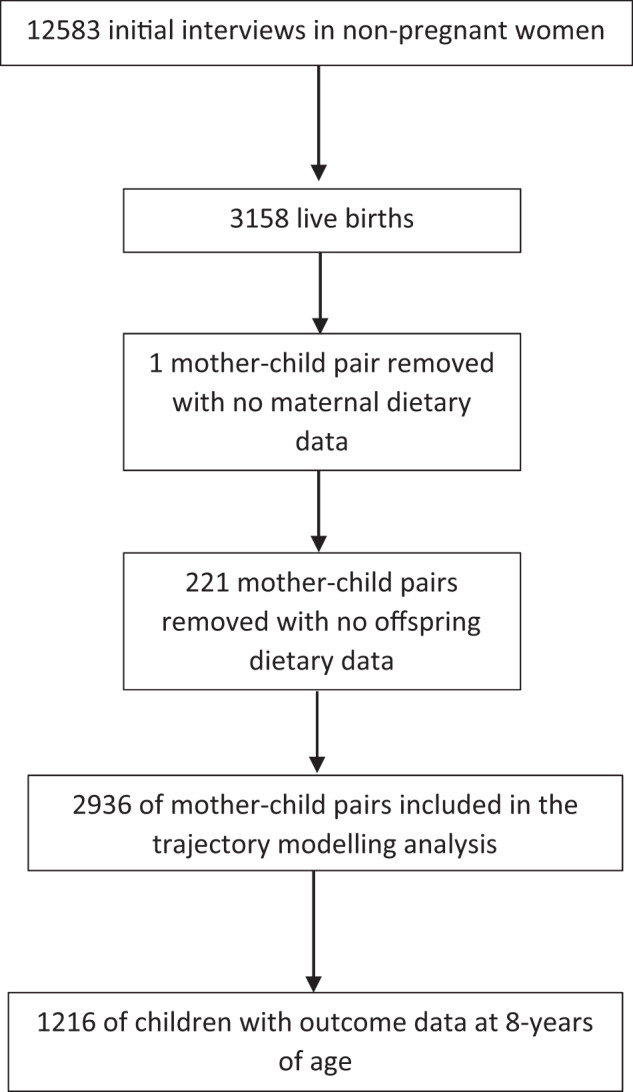
Flow diagram.

Figure 1 illustrates the flowchart of participants eligible for the analysis. 3158 mothers gave birth to live born infants in SWS; although GBTM can handle missing outcome data, we excluded mother-child dyads if the mother (*n* = 1) or the child (*n* = 221) were missing all of their dietary assessment points. Of the 2936 included in the GBTM, 1216 children provided adiposity outcomes at 8–9 years of age.

For the 2936 mothers included in the analysis, the median BMI at the preconception visit was 24.1 kg/m^2^ (21.8–27.3), 57% were categorised as having a healthy BMI, 2% were underweight and 41% had overweight or obesity. 59% had achieved a-levels or higher. Most (96%) of the cohort were White. Just under half (44%) were current or previous smokers, with 15% who smoked in pregnancy. Approximately half (48%) were multiparous and the mean age at birth was 30.7 years (Table 1). For the 1216 children who provided adiposity outcomes at 8–9 years of age (Supplementary Table 2), 62% were breastfed for >1 month. The median gestational age at delivery was 40.0 weeks, the average birthweight was 3447 g and 51% were female (Supplementary Table 2). In comparison with the SWS families who did not take part, mothers who attended the 8–9 year follow-up with their child were older, more likely to have achieved higher educational attainment, less likely to be a smoker and tended to have breastfed for longer (Supplementary Table 2).

**Table 1:** Demographic characteristics of 2936 mother-child pairs from the Southampton Women’s Survey.

Maternal		NMean (SD)/N (%)/Median (IQR)^a^		
Body mass index (kg/m^2^)			2910	24.1 (21.8–27.3)
Body mass index categories (kg/m^2^)	Underweight			49 (2%)
	Healthy weight			1658 (57%)
	Overweight			798 (27%)
	Obese			405 (14%)
Qualification level	A levels or higher		2928	1741 (59%)
Ethnicity (white)			2936	2812 (96%)
Ever smoked			2934	1283 (44%)
Ever smoked in pregnancy			2802	432 (15%)
Parity (Multiparous)			2934	1418 (48%)
Age at birth (years)			2936	30.7 (3.8)
*Family*				
Dominant social class	Non-manual^β^		2891	2382 (82%)
*Child*				
Breastfeeding	>1 month		2806	1733 (62%)
Gestational age at delivery (weeks)			2936	39.8 (1.8)
Birthweight (grams)			2909	3442 (548.0)
Sex (female)			2936	1405 (48%)

*IQR* Interquartile range, *N* number, *SD* standard deviation.

^a^Binary and categorical variables are presented using counts and percentages. The distribution of continuous variables was assessed using coefficients of skewness and then summarised by mean and standard deviation (SD) or median and interquartile range (IQR) where appropriate.

^β^Includes professional, management and technical and skilled non-manual vs. skilled manual, partly skilled and unskilled.

**Fig. 2:**
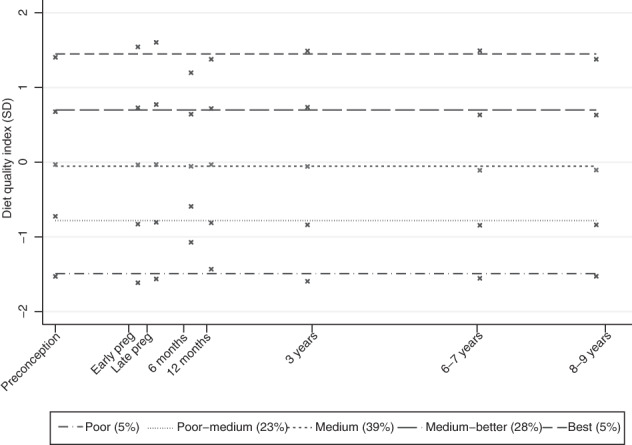
Diet quality trajectories, using group-based trajectory modelling, from preconception to 8–9 years of age.

A five-trajectory group model with intercept specification for all classes was identified as the optimal model. Classes 1–4 are illustrated in Supplementary fig. 3 and the model fit criteria for classes 1–6 are presented in Supplementary Table 3. Although the BIC was lower for the six classes, this model was rejected as only 1% of the population was assigned to one class. Figure 2 illustrates the dietary trajectories for the five groups with the 95% confidence interval based on predicted trajectory means and the individual trajectories are shown in Supplementary fig. 4. The diet quality trajectories were characterised as stable, horizontal lines, and were categorised as poor (*n* = 142, 5%), poor-medium (*n* = 667, 23%), medium (*n* = 1146, 39%), medium-better (*n* = 818, 28%) and best (*n* = 163, 5%).

Firstly, we examined the associations between early life factors and the diet quality trajectories, deriving OR across a five-category diet quality continuous variable (Table 2). Poorer diet quality was associated with higher maternal pre-pregnancy BMI [OR 1.05 (95% confidence interval (CI) 1.03, 1.06)], smoking in pregnancy [6.68 (5.45, 8.19)] and multiparity [2.63 (2.29, 3.01)], lower maternal age at birth [0.89 (0.87, 0.90)], lower educational attainment (all *p* < 0.001) and lower dominant social class (all *p* < 0.001). For the offspring, no breastfeeding or a short breastfeeding duration was associated with a poorer diet quality. Comparing characteristics of the poor and the best trajectory groups: 2% and 63% had degree qualifications, 55% and 2.5% smoked in pregnancy, 82% and 28% were multiparous and mean age at birth was 28.8 years and 31.9 years, respectively. There was no difference in the proportion of boys vs. girls across the groups.

**Table 2:** Maternal and child demographic characteristics according to the five diet trajectories obtained using group-based trajectory modelling (*n* = 2963).

Trajectory group		Poor (*n* = 142)	Poor-medium (*n* = 667)	Medium (*n* = 1146)	Medium-better (*n* = 818)	Best (*n* = 163)	Odds ratio (95% CI)^b^
Maternal		Mean (SD)/N (%)/Median (IQR)^a^					
Body mass index (kg/m^2^)		24.2 (22.0–27.6)	25.0 (22.2–28.9)	24.3 (22.1–27.4)	23.7 (21.7–26.3)	23.0 (21.2–25.6)	1.05 (1.03, 1.06)^***^
Body mass index categories (kg/m^2^)	Underweight	7 (5%)	13 (2%)	16 (1%)	10 (1%)	3 (2%)	2.11 (1.22, 3.64)^**^
	Healthy weight	76 (54%)	322 (49%)	637 (56%)	509 (63%)	114 (70%)	1
	Overweight	37 (26%)	194 (29%)	316 (28%)	213 (26%)	38 (23%)	1.30 (1.12, 1.52)^**^
	Obese	21 (15%)	131 (20%)	164 (14%)	81 (10%)	8 (5%)	1.96 (1.61, 2.38)^***^
Qualification level	A levels or higher	27 (19%)	228 (34%)	673 (59%)	668 (82%)	145 (90%)	0.19 (0.16, 0.22) ^***^
Ethnicity (white)		139 (98%)	655 (98%)	1098 (96%)	768 (94%)	152 (93%)	2.10 (1.52, 2.90)^***^
Ever smoked		99 (70%)	385 (58%)	480 (42%)	274 (33%)	45 (27%)	2.26 (1.97, 2.59)^***^
Ever smoked in pregnancy		77 (55%)	194 (30%)	128 (12%)	29 (4%)	4 (2%)	6.68 (5.45, 8.19)^***^
Parity (multiparous)		116 (82%)	428 (64%)	533 (47%)	295 (36%)	46 (28%)	2.63 (2.29, 3.01)^***^
Age at birth (years)		28.8 (4.2)	29.6 (4.1)	30.6 (3.7)	31.8 (3.4)	31.9 (3.2)	0.89 (0.87, 0.90)^***^
*Family*							
Dominant social class	Non-manual^β^	79 (55%)	475 (73%)	951 (84%)	729 (91%)	148 (91%)	0.34 (0.28, 0.41) ^***^
*Child*							
Breastfeeding	>1 month	26 (19%)	274 (42%)	694 (63%)	605 (79%)	134 (88%)	0.24 (0.21, 0.29) ^***^
Gestational age at delivery (weeks)		39.5 (2.0)	39.6 (1.9)	39.8 (1.8)	39.8 (1.8)	40.0 (1.4)	0.96 (0.92, 0.99) ^*^
Birthweight (grams)		3259.2 (572.9)	3445.5 (585.3)	3447.9 (537.8)	3457.3 (534.3)	3479.6 (477.6)	1.00 (1.00, 1.00)
Sex (female)		67 (47%)	299 (45%)	554 (48%)	412 (50%)	73 (45%)	0.90 (0.79, 1.03)

*IQR* Interquartile range, *N* number, *SD* standard deviation.

***p < 0.001; **p < 0.01; *P < 0.05.

^a^Binary and categorical variables are presented using counts and percentages. The distribution of continuous variables was assessed using coefficients of skewness and then summarised by mean and standard deviation or median and interquartile range where appropriate.

^b^Ordered logistic regression was used to assess the relationship between the demographic variables and the five trajectories on a continuous scale, the odds ratio refers to membership of a lower diet trajectory.

^β^Includes professional, management and technical and skilled non-manual vs. skilled manual, partly skilled and unskilled.

**Fig. 3:**
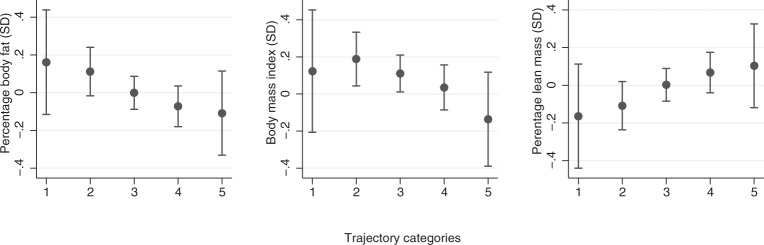
Adjusted means (and 95% CI) for offspring outcomes at 8–9 years of age. All outcomes were adjusted for maternal BMI, parity, highest maternal education attainment and maternal age. Percentage body fat and lean mass were also adjusted for offspring sex and age. Trajectory categories: 1 = poor, 2 = poor-medium, 3 = medium, 4 = medium-better, 5 = best.

Supplementary Table 4 reports the unadjusted associations between the dietary trajectories and adiposity outcomes at 8–9 years of age. A 1-class decrease in diet quality was associated with a higher BMI *z*-score, arm circumference, waist circumference and percentage body fat (all *p* < 0.001) and a lower height-for-age *z*-score and percentage lean mass (all *p* < 0.001). Following adjustment for maternal pre-pregnancy BMI, highest education attainment, age at birth and parity (Table 3) a one-class decrease in the diet quality trajectory was associated with higher percentage body fat [0.08 SD (0.01, 0.15)] and BMI *z*-score [0.08 SD (0.00, 0.16)] and a lower percentage lean mass [−0.08 SD (−0.14, −0.01)] (Table 3). Comparing the mean body fat percentage for the lowest diet trajectory with the highest (27.3% vs. 23.6%), there was a 14% difference between the two groups (Fig. 3). The loss of statistical significance from the unadjusted to the adjusted model illustrates the role of positive confounding between diet, socio-economic status and adiposity outcomes.

**Table 3:** Adjusted associations between the five dietary trajectories and adiposity *z*-scores at 8–9 year of age in children from the Southampton Women’s Survey.

Outcome	Poor (*n* = 47)	Poor-medium (*n* = 249)	Medium (*n* = 484)	Medium-better (*n* = 357)	Best (*n* = 79)	β-trend
	Regression coefficients (95% confidence interval)					
BMI for age^a^	0.26 (−0.16, 0.68)	0.33 (0.02, 0.63)^*^	0.25 (−0.03, 0.52)	0.17 (−0.10, 0.44)	Ref	0.08 (0.00, 0.16)^*^
Height for age^a^	0.07 (−0.31, 0.45)	−0.19 (−0.46, 0.09)	−0.05 (−0.30, 0.19)	0.05 (−0.19, 0.29)	Ref	−0.06 (−0.13, 0.01)
Weight for age^a^	0.17 (−0.23, 0.57)	0.10 (−0.18, 0.38)	0.12 (−0.14, 0.38)	0.13 (−0.13, 0.38)	Ref	0.01 (−0.06, 0.09)
Arm circumference	0.18 (−0.19, 0.54)	0.24 (−0.02, 0.50)	0.20 (−0.03, 0.44)	0.17 (−0.07, 0.40)	Ref	0.05 (−0.02, 0.11)
Waist circumference	0.23 (−0.14, 0.61)	0.14 (−0.12, 0.41)	−0.09 (−0.15, 0.33)	0.07 (−0.17, 0.30)	Ref	0.05 (−0.02, 0.11)
*DXA outcomes*						
Total body fat	0.26 (−0.13, 0.64)	0.18 (−0.10, 0.67)	0.13 (−0.13, 0.38)	0.09 (−0.16, 0.34)	Ref	0.06 (−0.02, 0.13)
Percentage body fat	0.27 (−0.10, 0.64)	0.22 (−0.05, 0.49)	0.11 (−0.13, 0.35)	0.04 (−0.20, −0.27)	Ref	0.08 (0.01, 0.15)^*^
Total lean	0.09 (−0.32, 0.50)	−0.01 (−0.30, 0.29)	0.12 (−0.15, 0.39)	0.18 (−0.10, 0.44)	Ref	−0.03 (−0.11, 0.04)
Percentage lean	−0.27 (−0.63, 0.10)	−0.21 (−0.48, 0.05)	−0.10 (−0.34, 0.14)	−0.04 (−0.27, 0.20)	Ref	−0.08 (−0.14, −0.01)^*^

All outcomes are adjusted for maternal pre-pregnancy BMI, maternal highest education attainment, maternal age at birth and parity. The DXA and circumferences outcomes have also been adjusted for child sex and age at the 8–9-year visit.

*BMI* Body mass index, *DXA* dual energy x-ray absorptiometry.

*p < 0.05.

^a^WHO *z*-scores were calculated using the WHO standards which are age and sex standardised [18]. The DXA and circumferences outcomes have been normalised using Fisher–Yates transformation. All regression coefficients represent the relative change in the standard deviation of the outcome per 1-category decrease in the diet trajectory.

## Discussion

This is the first study, to the authors’ knowledge that has applied a latent class modelling approach to dietary data collected from preconception to mid-childhood. This comprehensive analysis has described trajectories of diet quality across early life and explored associations between these trajectories and socio-demographic factors and adiposity outcomes at 8–9 years of age. We found that diet quality trajectories are stable from before pregnancy in the mothers to age 8–9 years in the child, and a poorer trajectory is associated with maternal socio-demographic factors including lower educational attainment, lower age at birth, higher BMI and smoking. Independently of maternal pre-pregnancy BMI, highest education attainment, age at birth and parity, a sustained poorer diet quality was associated with higher adiposity outcomes in the child at age 8–9-years.

Over recent years, several modelling approaches have been developed to identify longitudinal latent classes within a population [15]. These methodologies have frequently been applied to developmental [30] and early life growth data [36]. However, longitudinal dietary intake analyses tend to focus on average trajectories over time [37] and are normally limited by two- to three waves of data [38]. Previously in the SWS cohort, we have taken an approach to convert the continuous dietary indicator into thirds at each assessment point [28]. This categorical variable was then summed across all time points to create an overall diet quality score and showed that a poor diet quality tracks across early-life and continued exposure to a poor diet quality from 6 months to 6 years of age was associated with higher fat mass. We have advanced these prior analyses in this study by including maternal preconception and antenatal diet quality to investigate the earliest dietary exposures of the child. In addition, the analyses in the current study describe changes in diet quality over time, which offers new insights into identifying timepoints and subgroups for nutritional interventions.

Our findings clearly illustrate the relationships between several maternal socio-demographic characteristics and diet quality across early life. Family social class, maternal education, smoking status in pregnancy and no breastfeeding or a shorter breastfeeding duration showed the strongest associations with early life diet trajectory. These findings are consistent with existing evidence which shows that unhealthy lifestyle behaviours, such as smoking, poor nutrition and short breastfeeding duration are inversely associated with socioeconomic position [39]. Poorer offspring dietary patterns have also consistently been associated with lower levels of maternal educational attainment [40]. It is important to identify population groups at risk of poor diet quality, so that interventions during the lifecourse can be targeted appropriately [41]. An additional consideration, which is well documented in the literature, is the relationship between socioeconomic inequalities and clustering of unhealthy behaviours, including the food environment and the development of adverse health outcomes [42, 43]. Our findings support the need to enact effective and appropriate nutrition interventions to promote longer term health outcomes, particularly among families from socially deprived backgrounds [41].

Continued exposure to a poor diet quality from preconception to mid-childhood was associated with higher adiposity in the child. We also observed a 14% difference in percentage fat mass between the lowest and the highest dietary trajectories. Several cohort studies have reported relationships between poor diet quality in early life and measures of childhood obesity [44, 45]. Furthermore, there is evidence to suggest that dietary behaviours established in childhood track into adolescence and adulthood [46]. Viewed collectively, these research findings suggest that mothers who consume a diet of poorer quality are likely to have children who follow a poor diet quality trajectory into later life and are potentially at greater risk of developing overweight/obesity throughout the lifecourse. Therefore, our data support intervening in the earliest stages of the life-course, ideally before conception, to lower the risk of a child developing obesity [9, 47].

There is a growing awareness within the academic and public health communities of the impact maternal preconception health has on offspring outcomes [48]. Smoking status, alcohol intake and poor dietary intake, including inadequate micronutrient status, have all been associated with adverse maternal and childhood outcomes [9, 49]. However, despite this increasing awareness, recognition of the importance of the preconception period remains low amongst the general public and healthcare professionals [48]. Results from this study provide additional evidence of the tracking of diet quality from preconception through to mid-childhood. This observation highlights the need for public health interventions in the years and months leading up to conception. Policy measures which support improvements in diet quality across populations preconceptionally could have continued benefits across the antenatal period and impact on the offspring diet [50].

### Strengths and limitations

This study has several strengths, notably that the data presented are from a large longitudinal mother-child cohort with information collected from multiple assessment points across early life. SWS is currently the only European cohort where data have been collected prospectively in the mother before conception. The study includes comprehensive family, environmental and anthropometric data, as well as DXA as a recognised measure of adiposity [51]. Due to the detailed data collection, SWS provides an opportunity to explore the relationship between maternal and offspring dietary intake and the relationship with childhood adiposity. Limitations include attrition of the study population; this is a common feature of cohort studies and may result in selection bias and collider bias [52]. The observational study design is limited by residual confounding and may have resulted in over-estimation of reported effect sizes. In addition, dietary intake was assessed using FFQs; this type of methodology has been associated with recall bias [53]. A further limitation of the dietary analysis, for the SWS we used several different FFQs as these were age specific. Therefore, in our present analysis we used natural scores. However, future studies, if they use the same FFQ at all timepoints, would be able to use the applied scores which would have a constant score at each time point. In relating classes to outcomes, uncertainty in class membership has not been accounted for. This could be addressed using the BCH method [54] but a limitation of Stata is that the BCH method cannot be employed after a GBTM. A limitation of GBTM is that assumes no inter-individual differences in change within class, therefore the error variance is assumed to be the same for all classes and all time points [30]. Growth mixture modelling (GMM) is another latent class modelling technique which provides greater flexibility as it allows for a varying covariance structure within each class. As part of a sensitivity analysis, we compared the GBTM output for the five class model to the corresponding output using GMM and there was a strong agreement between these two methods (Spearman’s correlation = 0.98). Finally, we chose the ‘best’ group as the reference category as this group reflects a high diet quality which will aid interpretation of the findings. However this group does have a low participant number, and this may widen confidence intervals for the estimates for the other groups.

### Conclusion

Childhood obesity is arguably one of the biggest challenges for public health. Excess weight gain in early life has a significant impact on short and long-term health outcomes, including the development of type 2 diabetes, cardiovascular and lung disease and some forms of cancer. This work has shown that diet quality tracks from the preconception period in the mother to mid-childhood in the offspring, and a poorer diet quality was associated with lower family social class. Furthermore, continued exposure to a poor diet across early life was also associated with higher adiposity in the child. These findings have the potential to inform targeted public health strategies focused on reducing rates of childhood obesity; and include recommendations for providing families from socially deprived backgrounds with tailored interventions. Targeted strategies during the preconception period, alongside population interventions to improve diet more generally, may provide opportunities to promote positive dietary changes in the mother and subsequently the child, therefore reducing the risk of the child developing obesity in early life.

## Supplementary information

Supplementary file
