# Supplementary material for: Longitudinal dietary trajectories from preconception to mid-childhood in women and children in the Southampton Women’s Survey and their relation to offspring adiposity: a group-based trajectory modelling approach
Source: Int J Obes (Lond). 2021 Dec 16;46(4):758–66. doi: 10.1038/s41366-021-01047-2 (PMC8960403; doi:10.1038/s41366-021-01047-2)
Supplement: Supplementary file 1 — Supplementary file [file 41366_2021_1047_MOESM1_ESM.pdf]

Supplementary Tables and Figures

Supplementary Figure 1: DAG for child adiposity and lean mass

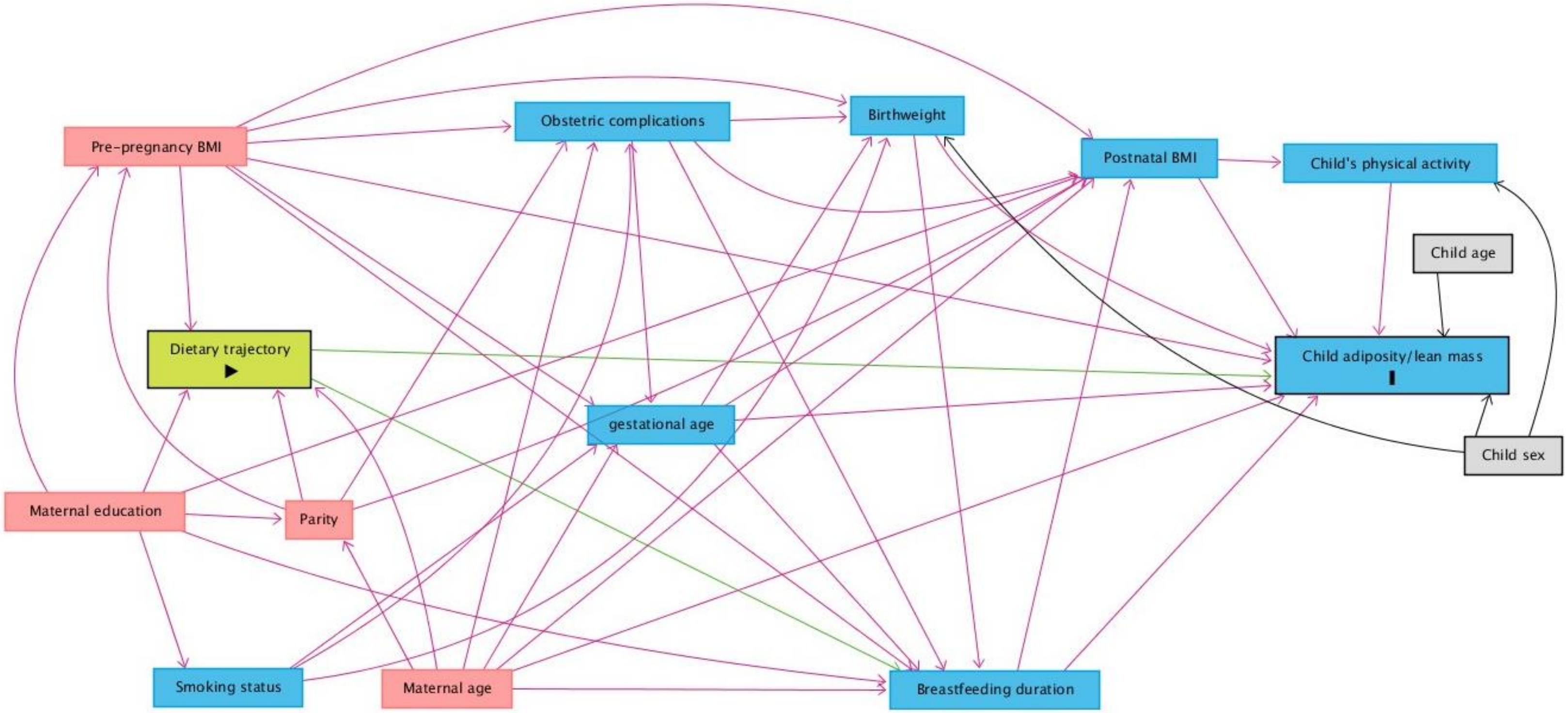

Supplementary Figure 2: DAG for child height

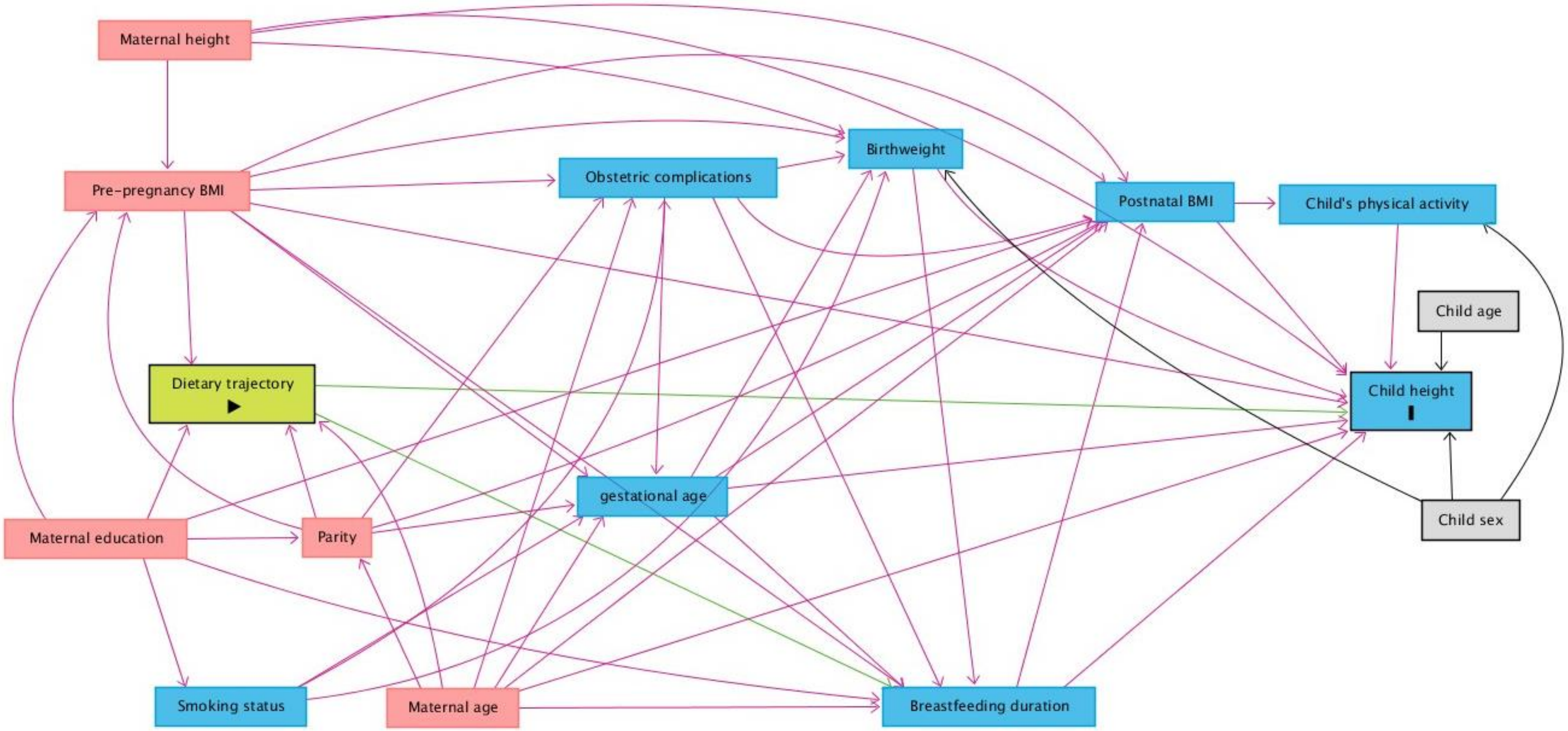

Supplementary figure 3: Group based trajectory modelling from one to four classes.

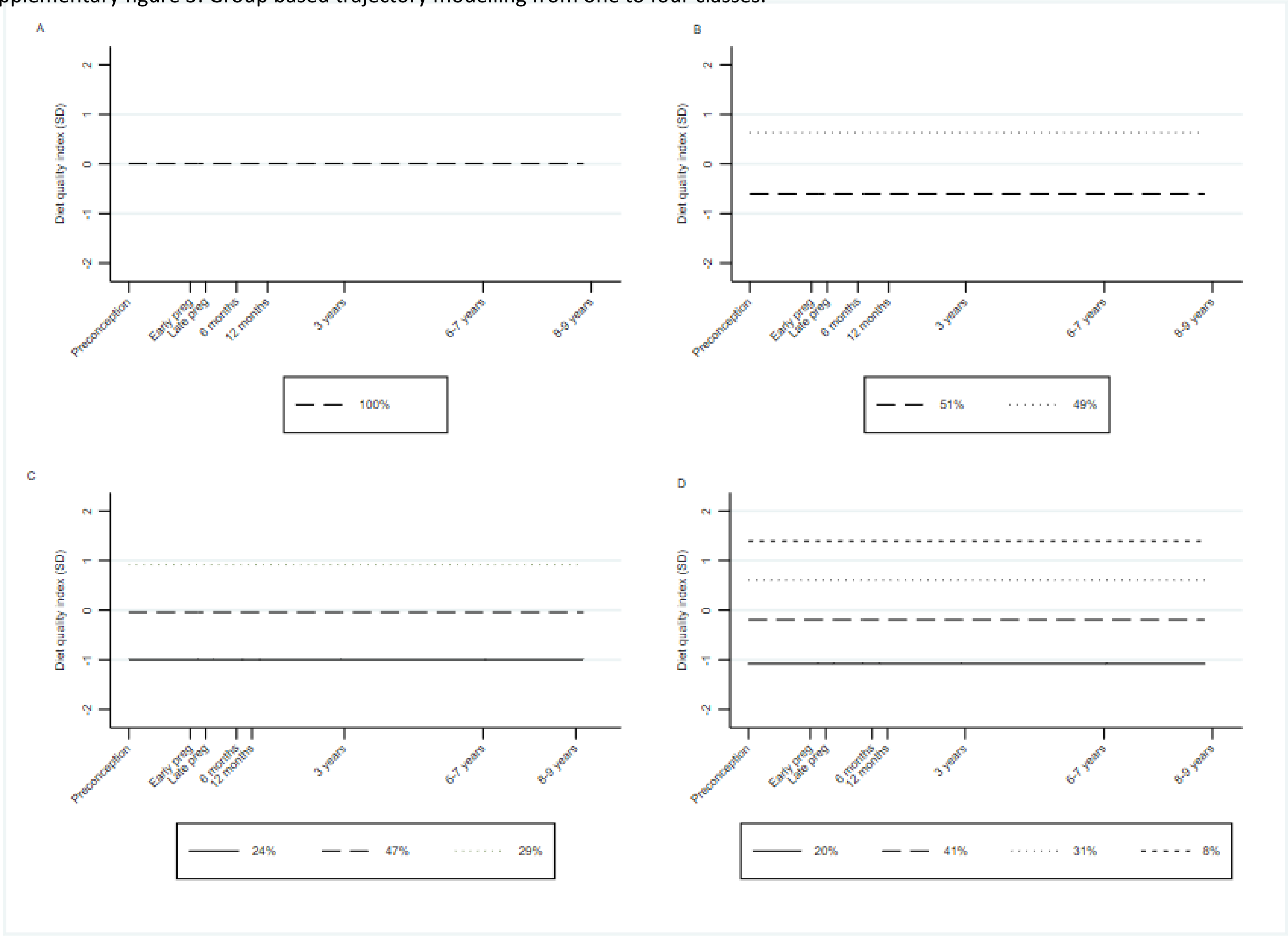

Supplementary figure 4: Individual trajectories for the 5 class group based trajectory model.

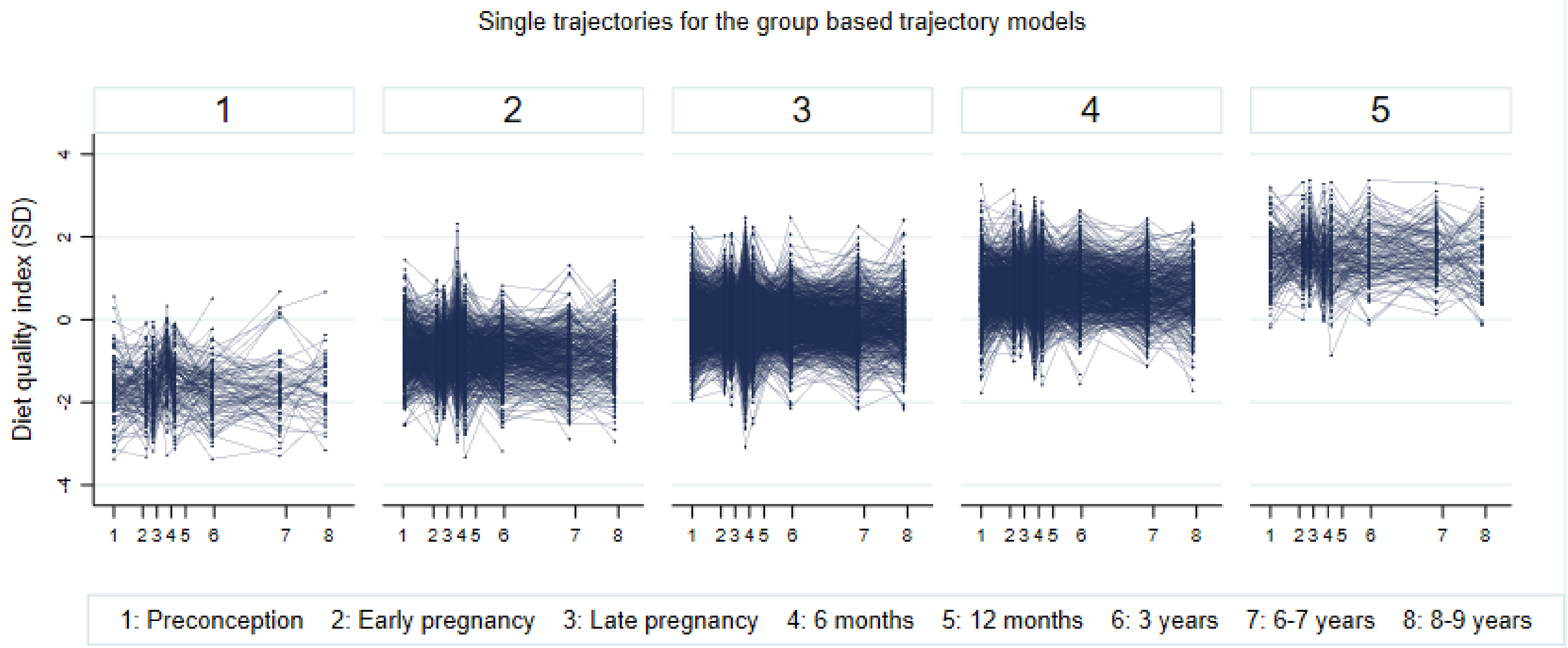

Supplementary Table 1: Guidelines for Reporting on Latent Trajectory Studies (GRoLT) checklist

Item 1: metric of time	Age in weeks from birth
Item 2: fixed or varying occasion	8 fixed time points, preconception, early pregnancy, late pregnancy, 6 and 12 months of age, 3, 6-7 and 8-9 years of age.
Item 3a: missing data mechanism	Missing at random, there was available data at the following timepoints: Preconception n= 3156, Early pregnancy n=2222, Late pregnancy n=2643, 6-months n=1869, 12-months n=2206, 3 years n=2625, 6-7 years n=2032 and 8-9 years n=1213.
Item 3b: auxiliary variables	Supplementary Table 1 reports demographic data of those lost to follow-up. Participants who completed the 8-9 appointment were more likely to have a higher education attainment, less likely to smoke, higher maternal age at birth, higher socioeconomic status and more likely to breastfeed for longer.
Item 3c: how dealt with missing data	If the mother (n=1) or the child (n=221) were missing all of their data points these participants were excluded from the analysis.
Item 4: distribution	Censored normal distribution
Item 5: software	Stata 15.0 'traj' command
Item 6a: LGMM versus LCGA	Our findings for the LCGA were compared with the output of a LGMM.
Item 6b: across-class variance-covariance matrix	Default
Item 7: functional form	Intercept function
Item 8: covariates	Covariates were not included in the model to predict trajectory groups
Item 9: random starts	Default
Item 10: model comparison	Average Posterior Probability Assignment, Bayesian Information Criterion, odds of correct classification, entropy, percentage of participants assigned to each group
Item 11: 1-class solution	Supplementary table 2
Item 12: sample size per class	poor (n=142, 5%), poor-medium (n=667, 23%), medium (n=1146, 39%), medium-better (n=818, 28%) and best (n=163, 5%).
Item 13: entropy	0.72-0.76
Item 14a: plot of final solution	Figure 2
Item 14b: plots for each model	Supplementary figure 3
Item 14c: plots of individual trajectories	Supplementary figure 4
Item 15: descriptive statistics	Table 1
Item 16: syntax	Supplementary file

Supplementary Table 2: Demographic characteristics of all SWS participants vs those who attended the 8-year follow-up

**Maternal**		**8-year follow-up n=1216**	**Didn't attend the 8-year follow-up n=1942**	**Total SWS cohort n=3158**	**follow-up (n=1216) vs those who did not (n=1942)** [b]
		**Mean (SD)/ Median (IQR) / N (%)** [a]			
Body mass index (kg/m^2)		24.2 (22.0-27.4)	24.1 (21.7-27.4)	24.1 (21.9-27.4)	0.65
Body mass index categories (kg/m^2)	Underweight	16 (1%)	36 (2%)	52 (2%)	0.21
	Healthy weight	679 (56%)	1106 (57%)	1787 (57%)	
	Overweight	346 (29%)	501 (26%)	850 (27%)	
	Obese	161 (13%)	280 (15%)	441 (14%)	
Qualification	A levels or higher	761 (63%)	1083 (56%)	1844 (59%)	<0.001
Ethnicity (white)		1170 (96%)	1846 (95%)	3016 (95%)	0.14
Ever smoked		470 (38%)	923 (48%)	1393 (44%)	<0.001
Ever smoked in pregnancy		142 (12%)	343 (19%)	485 (16%)	<0.001
Parity (Multiparous)		557 (46%)	986 (51%)	1543 (49%)	0.006
Age at birth (years)		31.0 (3.7)	30.4 (3.9)	30.6 (3.8)	<0.001
**Family**					
Dominant social class	Non-manual*	1038 (86%)	1508 (79%)	2546 (82%)	<0.001
**Child**					
Breastfeeding	> 1month	785 (67%)	980 (56%)	1765 (61%)	<0.001
Gestational age at delivery (weeks)		40.0 (39.0-41.0)	40.0 (39.0-40.1)	40.0 (39.0-41.0)	0.29
Birthweight (grams)		3447 (540.8)	3421 (576.8)	3431 (563.2)	0.21
Sex (female)		615 (51%)	905 (47%)	1,520 (48%)	0.04

Abbreviations: IQR: Interquartile range, N: number; SD: standard deviation. [a] *Binary and categorical variables are presented using counts and percentages. The distribution of continuous variables was assessed using coefficients of skewness and then summarized by mean and standard deviation or median and interquartile range where appropriate.* [b] *Differences between those who attended the 8-year follow-up vs those who did not was conducted by t-test or Mann–Whitney rank-sum test for continuous variables and χ2-test for categorical variables.* **includes professional, management and technical and skilled non-manual vs skilled manual, partly skilled and unskilled.*

Supplementary Table 3: Traj criteria statistics for 2 to 6 trajectories

	BIC	Group membership	APPA	Entropy	OCC
1 class	-24720	(1) 100%	-	-	-
2 classes	-22116	(1) 51%	(1) 0.94	0.80	18.8
		(2) 49%	(2) 0.94		18.4
3 classes	-21356	(1) 24%	(1) 0.90	0.78	34.7
		(2) 48%	(2) 0.89		10.1
		(3) 28%	(3) 0.91		30.0
4 classes	-21187	(1) 20%	(1) 0.88	0.76	34.5
		(2) 41%	(2) 0.86		10.2
		(3) 32%	(3) 0.84		12.6
		(4) 7 %	(4) 0.84		71.7
5 classes	-21098	(1) 5%	(1) 0.82	0.74	85.2
		(2) 23%	(2) 0.81		16.4
		(3) 39%	(3) 0.82		8.7
		(4) 28%	(4) 0.83		14.6
		(5) 5%	(5) 0.84		85.2
6 classes	-21094	(1) 1%	(1) 0.81	0.72	324.7
		(2) 9%	(2) 0.74		27.9
		(3) 20%	(3) 0.72		10.8
		(4) 37%	(4) 0.79		7.74
		(5) 27%	(5) 0.83		15.1
		(6) 5%	(6) 0.83		83.1

Abbreviations: APPA: Average Posterior Probability Assignment, BIC: Bayesian Information Criteria, OCC: odds of correct classification.

Supplementary table 4: Unadjusted association between the 5 dietary trajectories and adiposity z-scores at 8-9 year of age in children from the Southampton Women's Survey

Outcome	Poor (n=47)	Poor-medium (n=249)	Medium (n=484)	Medium-better (n=357)	Best (n=79)	β-trend
			**Regression coefficients (95% confidence interval)**			
BMI for age [a]	0.52 (0.10, 0.93) *	0.61 (0.32, 0.90) ***	0.46 (0.19, 0.73) **	0.30 (0.02, 0.58) *	Ref	0.15 (0.08, 0.22) ***
Height for age [a]	-0.02 (-0.38, 0.33)	-0.24 (-0.49, 0.01)	-0.09 (-0.32, 0.14)	0.04 (-0.20, 0.28)	Ref	-0.08 (-0.14, -0.02) **
Weight for age [a]	0.31 (-0.07, 0.69)	0.29 (0.02, 0.55) *	0.29 (0.01, 0.51) *	0.21 (-0.04, 0.47)	Ref	0.06 (0.00, 0.12)
Arm circumference	0.35 (-0.01, 0.71)	0.44 (0.19, 0.69) **	0.36 (0.12, 0.59) **	0.27 (0.03, 0.51) *	Ref	0.09 (0.03, 0.15) **
Waist circumference	0.37 (0.01, 0.73) *	0.33 (0.08, 0.58) *	0.23 (-0.01, 0.47)	0.16 (-0.08, 0.40)	Ref	0.09 (0.03, 0.15) **
**Dual energy x-ray absorptiometry outcomes**						
Total body fat	0.50 (0.11, 0.89) *	0.41 (0.12, 0.69) **	0.30 (0.03, 0.57) *	0.20 (-0.07, 0.48)	Ref	0.11 (0.05, 0.18) **
Percentage fat	0.51 (0.12, 0.90) *	0.42 (0.13, 0.70) **	0.27 (0.00, 0.54) *	0.14 (-0.13, 0.42)	Ref	0.13 (0.07, 0.20) ***
Total lean	0.19 (-0.20, 0.59)	0.15 (-0.14, 0.44)	0.22 (-0.05, 0.48)	0.25 (-0.03, 0.53)	Ref	0.00 (-0.07, 0.07)
Percentage lean	-0.51 (-0.90, -0.11) *	-0.41 (-0.69, -0.12) **	-0.26 (-0.52, 0.01)	-0.14 (-0.41, 0.14)	Ref	-0.13 (-0.19, 0.06) ***

BMI: Body mass index, DXA: dual energy x-ray absorptiometry.

[a] *WHO z-scores were calculated using the WHO standards which are age and sex standardized* [18].

The DXA and circumferences outcomes have been normalized using Fisher-Yates transformation. All regression coefficients represent the relative change in the standard deviation of the outcome per 1-class decrease in the diet trajectory. *** *p<0.001,* ** *p<0.01,* * *P<0.05.*

Example code: traj, var(zpc*) indep(t_*) model(cnorm) order(0 0 0 0 0) min(-4) max(4)
